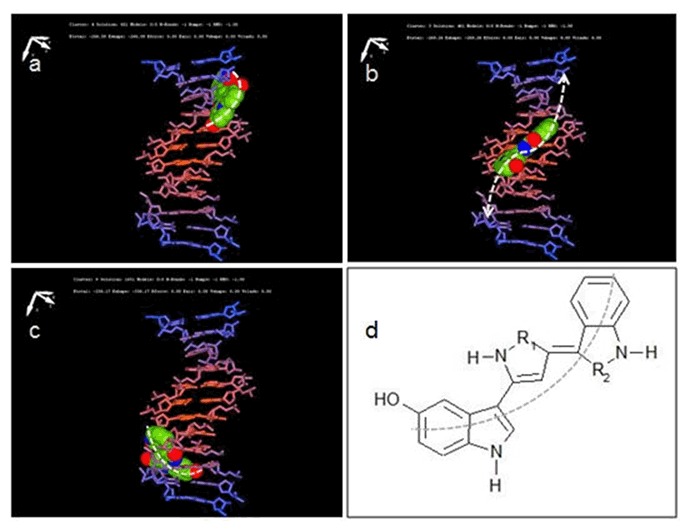# Antimicrobial activity of PVP from an Antarctic bacterium, *Janthinobacterium* sp. Ant5-2, on multi-drug and methicillin resistant *Staphylococcus aureus*

**DOI:** 10.1007/s13659-012-0021-4

**Published:** 2012-04-11

**Authors:** Jonathan P. Huang, Nazia Mojib, Rakesh R. Goli, Samantha Watkins, Ken B. Waites, Rasik Ravindra, Dale T. Andersen, Asim K. Bej

**Affiliations:** 1 Department of Biology, University of Alabama at Birmingham, Birmingham, AL 35294-1170 USA; 2 Department of Pathology, University of Alabama at Birmingham, Birmingham, AL 35294-1170 USA; 3 Head Land Sada, National Centre for Antarctic & Ocean Research, Vasco-da-Gama Goa, 403804 India; 4 Carl Sagan Center for the Study of Life in the Universe, SETI Institute, Mountain View, CA 94043 USA; 5 Red Sea Research Center, King Abdullah University of Science and Technology, Thuwal, 23955-6900 Saudi Arabia

**Keywords:** natural product, bacterial pigment, resazurin assay, minimum inhibitory concentration (MIC)

## Abstract

Multiple drug resistant (MDR) and methicillin-resistant *Staphylococcus aureus* (MRSA) have become increasingly prevalent as a community acquired infection. As a result limited treatment options are available with conventional synthetic antibiotics. Bioprospecting natural products with potent antimicrobial activity show promise for developing new drugs against this pathogen. In this study, we have investigated the antimicrobial activity of a purple violet pigment (PVP) from an Antarctic bacterium, *Janthinobacterium* sp. Ant5-2 on 15 clinical MDR and MRSA strains. The colorimetric resazurin assay was employed to determine the minimum inhibitory concentration (MIC_90_) of PVP against MDR and MRSA. The MIC_90_ ranged between 1.57 µg/mL and 3.13 µg/mL, which are significantly lower than many antimicrobials tested from natural sources against this pathogen. The spectrophotometrically determined growth analysis and total microscopic counts using Live/dead® *Bac*Light™ fluorescent stain exhibited a steady decrease in viability of both MDR and MRSA cultures following treatment with PVP at the MIC levels. *In silico* predictive molecular docking study revealed that PVP could be a DNA-targeting minor groove binding antimicrobial compound. The continued development of novel antimicrobials derived from natural sources with the combination of a suite of conventional antibiotics could stem the rising pandemic of MDR and MRSA along with other deadly microbial pathogens.

## Introduction

*Staphylococcus aureus* is an opportunistic human pathogen and recognized as the leading overall cause of hospital-acquired infections worldwide. It can remain asymptomatic as part of the normal flora on human skin for months before any form of infection manifests. Following infection, the mortality rates could reach over 80% if left untreated.[1] Although commonly associated with skin and soft tissue infections, bacteria entering the blood stream serve as a conduit for systemic infection, causing septic shock and severe damage to organs.[2] Despite advances in diagnosis and introduction of synthetic antibiotics, the available treatment options for *S. aureus* infections are severely limited due to a rise in antimicrobial resistant strains. The common antibiotic resistant phenotype present in both the hospital and community settings is methicillin resistance by this pathogen.[3] Reports of MRSA (Methicillin-Resistant *Staphyloccocus aureus*) first surfaced less than a year after the antibiotic was introduced in 1960 for this pathogen. Since then its prevalence has much increased in recent years.[4] For example in 1974, MRSA infections constituted 2% of the total number of staphylococcal infections; 30 years later, this number climbed to 67%.[5] In the United States alone, MRSA-related hospitalizations have increased from 127,036 in 1999 to 278,203 in 2005.[6] This bacterium has exhibited *in vitro* susceptibility to a wide variety of synthetic antibiotic classes, including Trimethoprim-Sulfamethoxazole, Erythromycin, Tetracycline, and Linezolid.[7–9] Due to the rapid adaptation against current antibiotics, *S. aureus* has become resistant to almost all classes of antibiotics leading to the prevalence and spread of multiple drug resistant (MDR) strains.[10,11] Therefore there is a necessity to introduce new antimicrobials as treatment regimens against the MDR and the

*To whom correspondence should be addressed. E-mail: abej@uab.edu

MRSA strains. In this pursuit, the antibacterials from natural sources that can suppress MRSA infections without the consequence of developing antibacterial resistance are desirable.[12] Natural products such as tea tree oil, sanguinarine, betulinic acid, and chinese *Qingre* granules have been shown to exhibit inhibitory activity on MRSA strains.[7,13–16] An extract of a Ghanaian plant, *Alchornea cordifolia* exhibited antibacterial activity against 21 bacterial pathogens including *Streptococcus pyogenes*, *Escherichia coli*, *Pseudomonas aeruginosa*, *Proteus vulgaris,* MRSA and MSSA (methicillin sensitive *S. aureus*).[17] Many bacteria inhabiting Antarctica under extreme cold, dry and high solar UV radiation conditions produce secondary metabolites, some of which have been shown to inhibit the growth of other bacteria including human pathogens.[18] Therefore bioprospecting secondary metabolites from these Antarctic extremophiles have the potential for the discovery of effective antimicrobials particularly against MDR pathogens. In a recent study, 22 out of 580 bacterial strains from soil and marine samples across the Terra Nova Bay in Antarctica exhibited antimicrobial activity against *Escherichia coli*, *Proteus mirabilis*, *Micrococcus luteus*, and *Bacillus subtilis*.[19] Previously we have reported the antibacterial activity of the purple violet pigment (PVP) from an Antarctic bacterium *Janthinobacterium* sp. Ant5-2 on virulent and avirulent strains of *Mycobacterium tuberculosis*.[20] In this study we describe the efficacy of PVP as an antimicrobial for MDR and MRSA strains of *S. aureus* and a comparison of the MICs of PVP with previously reported other antimicrobials from natural sources as well the synthetic antibiotics. Further, we predicted the possible mechanism of action of PVP using computational biology and bioinformatics approach.

## Results and Discussion

An increasing trend of the MDR and MRSA-related infections and mortality in humans with limited options of treatment using conventional antibiotics led to the identification of novel antimicrobial compounds that are derived from natural sources.[15,21] In a previous study in our laboratory, we purified PVP from an Antarctic bacterium, *Janthinobacterium* sp. Ant5-2 and demonstrated the antimycobacterial activity on *M. smegmatis* and MDR and XDR strains of *M. tuberculosis*.[20] Furthermore Mojib et al. elucidated the chemotherapeutic activity of PVP against skin cancer cells without any adverse effect on normal human fibroblasts or keratinocytes.[22] We also found that PVP did not cause hemolysis of red blood cells when plated in blood agar medium (unpublished data). In this report we present the effectiveness of PVP on MDR and MRSA strains by determining the MICs using resazurin assay. Resazurin, a blue non-fluorescent dye, is reduced by live cells into a pink colored and highly fluorescent resorufin.[23,24] This assay was previously utilized to determine the viability of drug-resistant strains of *Mycobacterium* against different antibiotics.[25–27] We have used the resazurin assay instead of the traditional agar diffusion or agar dilution methodologies because PVP is a nonpolar compound, which generally diffuses at a slower rate than polar compounds in agar medium thus providing an erroneous result. Moreover, the agar diffusion assay provides the qualitative information while the resazurin assay is suitable for both qualitative and quantitative analyses. Meanwhile the agar dilution methodology necessitates large amounts of a test compound (e.g., PVP), which is generally not attainable with extracts from natural products.[28]

The resazurin assay determined $MIC_{90}$ of PVP on type strain and the clinical strains of *S. aureus* which are listed in Table 1. All 15 clinical isolates of *S. aureus* had positive amplification of the *Sa442* gene while the methicillin-resistant gene, *mecA*, was identified in 3 clinical strains (10.005, 10.007, and 10.014). The $MIC_{90}$ were determined by < 10% percent survival of cells after treatment with PVP. Overall the effectiveness of PVP on conventional drug-resistant strains of *S. aureus* ranged from 1.57 $\mu$g/mL to 6.25 $\mu$g/mL including MRSA strains ($MIC_{90}$ ranged from 1.57 $\mu$g/mL to 3.13 $\mu$g/mL) (Table 1; Figure 1). Interestingly the control antibiotic

**Table 1. List of clinical and type strains of *Staphylococcus aureus* strains used in this study. The minimum inhibitory concentrations (MICs) of PVP are listed.**

| *Staphylococcus aureus* strains | Source[b] | Antibiotics Resistant to | $MIC_{90}$ ($\mu$g/mL)[a] |
|---|---|---|---|
| *S. aureus* ATCC 25923 | Clinical | Sensitive to all drugs | 6.25 |
| 10.004 | Tissue | Ampicillin, Penicillin | 3.13 |
| 10.005 | Blood | Amoxicillin/Clavulanic acid, Ampicillin, Ceftriaxone, Ciprofloxacin, Clindamycin, Erythromycin, Gentamicin, Levofloxacin, Methicillin, Oxacillin, Penicillin | 3.13 |
| 10.006 | Tissue | Sensitive to all drugs | 6.25 |
| 10.007 | Bone | Amoxicillin/Clavulanic acid, Ampicillin, Ceftriaxone, Ciprofloxacin, Clindamycin, Erythromycin, Gentamicin, Levofloxacin, Methicillin, Oxacillin, Penicillin, Trimethoprim/Sulfamethoxazole | 1.57 |
| 10.008 | Sinus | Ampicillin, Erythromycin, Penicillin | 3.13 |
| 10.009 | Sputum | Clindamycin, Erythromycin | 3.13 |
| 10.010 | Sinus | Ampicillin, Penicillin | 3.13 |
| 10.011 | Blood | Ampicillin, Erythromycin, Penicillin | 3.13 |
| 10.012 | Blood | Erythromycin, Penicillin, Trimethoprim/Sulfamethoxine | 3.13 |
| 10.013 | Tissue | Clindamycin, Erythromycin | 3.13 |
| 10.014 | Blood | Ampicillin, Ciprofloxacin, Clindamycin, Erythromycin, Levofloxacin, Methicillin, Penicillin, Trimethoprim/ Sulfamethoxazole | 1.57 |
| 10.015 | Tissue | Ampicillin, Ciprofloxacin, Levofloxacin | 3.13 |
| 10.016 | Blood | Clindamycin, Erythromycin, Tetracycline | 1.57 |
| 10.017 | Sinus | Clindamycin, Erythromycin | 3.13 |
| 10.018 | Sinus | Clindamycin, Erythromycin | 3.13 |
| 10.019 | Abdomen | Ampicillin, Ciprofloxacin, Levofloxacin, Penicillin, Tetracycline, | 1.57 |

[a]Minimum Inhibitory Concentrations (MICs) in $\mu$g/mL was determined by resazurin assay where < 10% of cells survived after treatment with PVP; [b]The origin of all strains was from human.

**Figure 1.** Effect of different concentrations of PVP on three clinical *S. aureus* strains (strain 10.005, 10.006 and 10.007) using resazurin assay. Asterisks denote the MICs of PVP for each clinical strain. MICs were determined at the concentration that showed < 10% survival after addition of pigment. Strains 10.005 and 10.007 were clinical MDR and MRSA isolates while the clinical isolate 10.006 is sensitive to antibiotics and served as control. These three strains were chosen since the MIC of each of the clinical strains fell within a similar range (1.57 $\mu$g/mL to 3.13 $\mu$g/mL). The standard error bars were obtained from three independent experiments.

sensitive strain 10.006 had a higher MIC (6.25 $\mu$g/mL) than the clinical MDR and MRSA strains 10.005 and 10.007 (Figure 1). Although it is unclear as to why the MDR and MRSA strains are more susceptible to PVP than the MSSA strain, one can predict that certain evolutionary adaptations that allowed these strains to become resistant to different classes of synthetic drugs may have caused them to become more vulnerable to PVP.[29,30] Due to the similar MIC of PVP on different clinical strains of *S. aureus*, we chose three strains (10.005, 10.006, and 10.007) to conduct further experimentation such as growth analysis and microscopic direct count.

We further investigated the MIC of PVP on the growth profiles (Figure 2) of the MDR and MRSA strains. The growth analysis of three clinical *S. aureus* strains (10.005, 10.006, and 10.007) are shown in Figure 2. A uniform decrease in cell viability and cell number with increased time of treatment of PVP was observed. The growth of each of the untreated (control) clinical strains reached stationary phase by the $4^{\text{th}}$ h. However the cultures treated with the PVP at its MIC concentration exhibited growth inhibition throughout the experimentation (Figure 2). By the $6^{\text{th}}$ h, the ratio between the optical density of the untreated and the PVP-treated clinical strains were 7.93% for strain 10.005, 10.84% for strain 10.006, and 8.51% for strain 10.007 (Figure 2).

We also analyzed the effect of PVP on MDR and MRSA strains through Live/Dead *Bac*Light total microscopic counts (Figure 3). Prior to treatment with PVP, *S. aureus* clinical strains (10.005, 10.006, and 10.007) displayed green fluorescence indicating live cells. After addition of PVP to *S. aureus* strain 10.005, 63.29% of the cells were viable 1h after treatment as compared to 8.46% after 6 h of treatment. For *S. aureus* strain 10.006, the viability decreased from 58.54% in

**Figure 2.** Growth curves of clinical strains of *S. aureus* with or without PVP treatment. Bacterial cultures were grown to $OD_{600}$= 0.2 before hourly spectrophotometric readings were documented. The bacterial strains were treated with the concentration of PVP corresponding to their respective MICs (strain 10.005 = 3.13 $\mu$g/mL; strain 10.006 = 6.25 $\mu$g/mL; strain 10.007 = 1.57 $\mu$g/mL) as determined by the resazurin assay. These three strains were chosen as a representative of the clinical *S. aureus* strains in this study. The standard error bars were obtained from three independent experiments.

1h after PVP treatment to 9.85% after 6 h of treatment. Finally, the viability of the *S. aureus* strain 10.007 decreased from 62.39% to 7.85% after 6 h of treatment (Figure 3). The $6^{\text{th}}$ h time course of PVP treatment exhibited decreased viability of the MRSA cultures, indicating a consistent inhibitory effect of PVP. Furthermore within the 6 h time period, the cell viability

**Figure 3.** Fluorescent microscopic direct counts of viable cells using Live/dead® *Bac*Light™ Bacterial Viability Kit (Molecular Probes, Inc). *S. aureus* clinical strains were treated with PVP at their respective MICs (strain 10.005 = 3.13 $\mu$g/mL; strain 10.006 = 6.25 $\mu$g/mL; strain 10.007 = 1.57 $\mu$g/mL). Due to similar results between the clinical strains of *S. aureus*, only the results of these three clinical strains are shown. Microscopic counts were conducted hourly with three replicates. The data presented are averages and standard error bars obtained from three replicates.

dropped under 10% (Figure 3).

The results from both growth profile and the microscopic counts correspond with the resazurin assays where low concentrations of PVP can cause inhibitory effects on this pathogen (Table 1 & Figure 1). We compared the efficacy of PVP with other reported natural antimicrobials and conventional antibiotics on MRSA strains (Table 2). The data observed in this study show higher effectiveness on MDR and MRSA strains than most other natural products, with the exception of tea-tree oil (2 $\mu$g/mL) and rhodomyrtone (0.5–2 $\mu$g/mL), which had similar effectiveness to PVP. Likewise, PVP has comparable effectiveness with many of the conventional antibiotics used to treat patients with MRSA infection. Moreover unlike PVP, some of these conventional drugs may cause adverse side effects to the patients.[31,22]

Though the mechanism of action of PVP is unclear, other studies have suggested that similar structural compounds (compounds consisting of bis-indole rings) may inhibit DNA or RNA synthesis or antibiotic resistance gene function by binding to the minor grooves of DNA but no experimental evidences have been reported.[48,49] In this study, we tested the above hypothesis by performing *in silico* binding analysis of PVP with B-DNA. The binding complexes between PVP and B-DNA were generated via docking (*Hex* 6.3) software (Fig. 4 a–c). Their relative stabilities were evaluated using molecular dynamics and their binding affinities, using free energy simulations. The highly stable nine clusters or complexes generated by *Hex* on docking produced E-total and E-shape (interaction free energy values) in the range of –297.14 to –258.17. It was predicted that the highly stable complexes were formed when PVP was bound to the minor grooves of B-DNA. Drug-DNA interactions can be either intercalation or groove binding.[50] Unlike DNA intercalators, DNA groove-binding molecules do not induce large conformational changes in the DNA but their binding is similar to standard lock- and key-models for ligand-macromolecular interactions.[51,52] It is also reported that the DNA groove binding molecules are usually crescent-shaped that interact across the minor grooves of B-DNA.[50] Based on the above assumptions and crescent-shape of PVP (Fig. 4d), our *in silico* data suggests that PVP could be a DNA minor groove binding compound and may implicate direct effects on DNA in the cell. This assumption can also be supported by a study where inactivation of the chromosomal copy of the *norA* gene in MDR *S. aureus*, which protects this pathogen from various classes of antibiotics such as quinolones and amphipathic cations, showed a 5- to 30-fold increase in drug susceptibility.[29] The *norA*-encoded NorA protein is necessary to pump out the antibiotics across the cell wall. Therefore it is possible that PVP, which is an alkaloid natural product, binds to MDR genes thereby inactivating the multi-drug resistance pumps. However further study is necessary to determine the exact mechanisms by which PVP functions as an effective antimicrobial on MDR and MRSA strains.

In conclusion, we have demonstrated that PVP, which is a bioactive natural product from an Antarctic bacterium *Janthinobacterium* sp. Ant5-2 causes growth inhibition of MDR and MRSA clinical strains. Relative to many traditional antibiotics and natural products, PVP exhibited a stronger efficacy on this pathogen. This study is the first to highlight the antibacterial effects of an Antarctic bacterial pigment on MDR and MRSA strains. The low concentration of PVP needed for bioactivity against MDR and MRSA may have an impact on developing future antibiotics. Further analysis of the mechanism of PVP along with *in vivo* study is necessary to assess its clinical utility.

**Figure 4.** Predictive molecular modeling of PVP bound to the minor grooves of B-DNA using *Hex* 6.3 docking software. The three different binding complexes represented by clusters are shown; cluster 4, E-total: –266.58 (**a**), cluster 3, E-total: –269.26 (**b**), cluster 9, E-total: –258.17 (**c**), where the stick model represents B-DNA and PVP molecule is represented by space-filled model. (**d**) The crescent-shaped molecular structure of PVP (3-[5-(3-hydroxyl-1H-indol-3yl)-2-$R_1$-1H-pyrrol-3-ylidene]-2-$R_2$-1H-indol)[20,22] also supports that this compound could be a groove binder.

## Experimental Section

**Bacterial Strains and PCR Amplification**. *Janthinobacterium* sp. Ant5-2 was isolated from a proglacial Lake Podprudnoye (also known as Lake P9) (S70°45'52.3" E11°37'10.7") located in Schirmacher Oasis, East Antarctica.[20] The bacterial strain was grown and maintained on R2A (BD Diagnostics, Sparks, MD) and Tryptic Soy Broth (TSB) (Remel, Lenexa, KS) at 15 ˚C. Reference type strain methicillin sensitive *S. aureus* (MSSA) ATCC 25923 and 15 clinical *S. aureus* strains were grown and maintained on TSB (Remel) and Tryptic Soy Agar (TSA) at 37 ˚C. Antibiotic resistance profiles of the *S. aureus* strains were determined by broth microdilution assay. The sources of these clinical strains of *S. aureus* are listed in Table 1.

Besides broth microdilution assay, the methicillin-resistance phenotype of the strains 10.005 and 10.007 were verified by PCR amplification of the *mecA* and the *sa442* genes. We used 2 sets of oligonucleotide primers reported previously: the first set of primers *Sa442F* (5'-GTCGGTACACGATATTCTTCAC G-3') and *Sa442R* (5'-CTCTCGTATGACCAGCTTCGGTAC-3') amplified a 179 bp amplicon; and the second set of primers, *mecA-F* (5'-CAAGATATGAAGTGGTAAATGGT-3') and *mecA-R* (5'-TTTACGACTTGTTGCATACCATC-3') amplified a 408 bp amplicon.[53–55] The *mecA* gene confers the methicillin-resistance while the *sa442* gene is a unique marker for *S. aureus*. Total genomic DNA from the *S. aureus* strains were

**Table 2. Minimum inhibitory concentration (MIC) for natural products and conventional antibiotics reported against Methicillin-resistant *Staphylococcus aureus* (MRSA)**

| Compounds | MIC ($\mu$g/mL) against MRSA[a] strains | References |
|---|---|---|
| *Natural products* | | |
| a-amyrin | 64 | [15] |
| betulinic acid | 64 | [15] |
| betulinaldehyde | 512 | [15] |
| Calozeloxanthone | 8.3 | [32] |
| Allicin | 16–32 | [33] |
| Tea-tree oil | 2 | [23] |
| Violacein | 5.1 | [34] |
| Sanguinarine | 3.13–6.25 | [14] |
| *Cinnamomum iners* standardized leaf extract | 100 | [35] |
| Propolis | 10 ($MIC_{50}$) | [36] |
| Enhydrin | 125–500 | [37] |
| *Rhodomyrtus tomentosa* leaf extract | 31.25–62.5 | [38] |
| Rhodomyrtone | 0.5–2 | [38] |
| Leguminosae medicinal plants | 100–500 | [39] |
| roots of *Atractylodes japonica* | 4–32 | [40] |
| essential oil of *Kadsura longipedunculata* | 60 | [41] |
| *Nigella sativa* | 200–500 | [42] |
| *Conventional antibiotics* | | |
| Vancomycin | ≤1–4 | [43]; [44] |
| Tetracycline | 4 | [45] |
| Trimethoprim/Sulfamethoxazole | 2.38 | [45] |
| Clindamycin | 8 | [46]; [44] |
| Chloramphenicol | 16 | [44] |
| Ciprofloxacin | 4 | [44] |
| Ceftriaxone | 4 | [44] |
| Linezolid | 2–3 | [47] |
| Teicoplanin | 4–8 | [47] |
| Purple violet pigment (PVP) from Antarctic *Janthinobacterium* sp. Ant5-2 | | |
| PVP | 1.57–3.13 ($MIC_{90}$) | This study |

[a]MIC levels are not indicated in the literature except those denoted with $MIC_{50}$ and $MIC_{90}$.

purified by alkaline lysis method.[56] The PCR parameters are as follows: 0.5 $\mu$g purified genomic DNA, 0.5 $\mu$M of each of the primers, 200 $\mu$M of each of the dNTPs, 1 × PCR buffer, 2.5 U of thermostable DNA polymerase (GenScript USA, Inc., Piscataway, NJ). The PCR thermal cycling parameters used are as follows: initial denaturation at 95 °C for 2 minutes followed by 30 cycles of amplification of the targeted gene, which consisted of denaturation at 95 °C for 30 sec, primer annealing at 50 °C for 30 sec, and primer extension at 72 °C for 40 sec. A final extension step was held at 72 °C for 10 minutes. The correct amplicon size was confirmed by agarose gel electrophoresis.

**Purification and Analysis of Bacterial Pigment.** The PVP from *Janthinobacterium* sp. Ant 5-2 was purified by liquid chromatography reverse phase flash column (C18 stationary phase; carbon 23%; particle size 40–60 lm, methanol/water (75:25) as mobile phase) and the fractions were combined and concentrated on a rotoevaporator.[57] After HPLC purification the solvents were removed and dried under high vacuum. Mass spectroscopy data were collected using the Micromass Electrospray Ionization Mass Spectrometer and an HP 1100 LC Micromass Platform LCZ with a C18 column. Additionally, the PVP was analyzed by Mass spectra and proton $^1$H NMR (Bruker ARX 700 spectrometer). The absorbance spectrum and concentration (OD = 575 nm; molar extinction coefficient 'ε' = 0.05601 mL/$\mu$g/cm) of PVP was recorded using Lambda2 UV/VIS Spectrometer (Perkin-Elmer) as described earlier.[20]

**Minimum Inhibitory Concentration (MIC).** *Resazurin Assay* The minimum inhibitory concentration (MIC) of PVP was determined by a colorimetric resazurin assay modified after Sarker et al.[58] Briefly, *S. aureus* cultures (5 mL) were grown to an $OD_{600}$ of 0.2–0.5. Aliquots (10 $\mu$L each) of the culture mixed in 50 $\mu$L of TSB were dispensed into a 96-well plate (Fisher Scientific). Purified PVP in 10% (v/v) DMSO (50 $\mu$L at two-fold concentration) was serially diluted; an additional 30 $\mu$L TSB was added into all the wells to achieve a final volume of 90 $\mu$L. The 96-well plate was then incubated at 37 °C overnight with agitation at 140 rpm. After 4 hours of incubation, 10 $\mu$L of resazurin (MP Biomedical) was added to each well. Control wells with only bacterial cultures did not receive PVP treatment, while growth medium control wells consisted of growth medium and PVP, but no bacterial culture. All resazurin assay data presented in this study were normalized by subtracting the background readings from an average of the growth medium control with PVP to avoid any interference of the colored PVP compound[20]. Fluorescence was measured at 530/590 nm using a microplate reader (DynaTech MR5000). For colorimetric evaluation, the microtiter wells with a color change from purple to pink or colorless signified as positive bacterial growth while wells remained purple signified no growth. The plates were prepared in triplicates.

**Antibacterial Activity of PVP.** *Growth Analysis* Spectrophotometric method ($OD_{600}$) was conducted to analyze the growth pattern of different clinical strains of *S. aureus* with or without PVP. *S. aureus* strains were grown in TSB at 37 ºC until the $OD_{600}$ reached to 0.2. Then the cultures were split into two aliquots: one aliquot was treated with PVP at the respective

MIC level and the other sample kept as untreated control. The bacterial growth of the PVP-treated and untreated cultures were monitored in a Lambda II spectrophotometer (Perkin Elmer) and optical density was recoded for 6 h. The experiments were conducted in triplicate samples.

*Fluorescence Microscopy* Live/dead® *Bac*Light™ Bacterial Viability Kit (Molecular Probes, Inc.) was used to fluorescently stain *S. aureus* cultures before and after treatment with PVP at the respective MICs.[59,60] The microscopic direct counts were conducted under a Lietz™ Diaplan epifluorescent microscope. In this assay, the cells with green fluorescence (SYTO 9) represented live cells while red fluorescence (propidium iodide) indicated dead cells. Cells were counted immediately after treatment with PVP and hourly thereafter for 6 hours. Approximately 350 ± 75 cells were counted for each time period for all three clinical strains. All experiments were conducted in triplicates.

*In Silico Binding Analysis* The B-DNA-PVP docking was done using *Hex* 6.3 software following the instructions on its online manual (http://hex.loria.fr/manual/hex_manual.html).[61] *Hex* is an interactive molecular graphics program that calculates and displays feasible docking modes of pairs of molecules using their 3D shapes information and spherical polar fourier (SPF) correlations. Since, *Hex* reads molecular structures from PDB-format files: the structure of B-DNA was retrieved from the Protein Data Bank (PDB) (PDB ID: 453D; DNA- benzimidazole complex). The ligand benzimidazole was removed from the original file to obtain the PDB file of the B-DNA. For PVP structure, the SDF (Structure Data Format) file comparable to that of violacein (CID 9928039) was retrieved from the PubChem Compound database. Then, this SDF file was converted to PDB format using Frog v1.01- FRee Online druG conformation generation software (http://bioserv.rpbs.univ-paris-diderot.fr/cgi-bin/Frog).[62] Finally, the modifications were incorporated in the PDB file according to the available structure information of PVP in our previous studies.[20,22]

**Acknowledgments**

We thank Col. (IL) J.N. Pritzker ARNG (Retired), Tawani Foundation (Chicago) for supporting the Tawani 2008 International Antarctic Scientific Expedition; Marty Kress, VCSI, Inc./NASA; Richard Hoover, NASA; and 2008–2009 Antarctic Maitri (India) and Novolazarevskaya (Russia) Station staffs.

**Open Access** This article is distributed under the terms of the Creative Commons Attribution License which permits any use, distribution, and reproduction in any medium, provided the original author(s) and source are credited.
